# Monthly or Weekly Supplementation with Cholecalciferol 20,000 IU in People Living with HIV: Results from a Nested Cohort Study

**DOI:** 10.1155/2018/7502127

**Published:** 2018-09-02

**Authors:** Sebastian Noe, Christine I. Moeckel, Christiane Schwerdtfeger, Celia Oldenbuettel, Hans Jaeger, Eva Wolf, Christoph D. Spinner

**Affiliations:** ^1^MVZ Karlsplatz, Research and Clinical Care Center, 80335 Munich, Germany; ^2^University Hospital Klinikum rechts der Isar, Department of Medicine II, Technische Universität München, 80675 Munich, Germany; ^3^MUC Research, Private Research Organization, 80335 Munich, Germany

## Abstract

**Background:**

There is still considerable uncertainty in handling vitamin D deficiency in people living with HIV (PLWH), due to a lack of comparative data and the wide range of recommended daily intake. Nondaily supplementation might be preferred in many PLWH, but recommendation on dosing has not been established. We aimed to compare the efficacy of weekly versus monthly supplementation with cholecalciferol 20,000 IU in a group of PLWH with vitamin D deficiency in Western Europe.

**Study Design:**

Longitudinal, retrospective nested cohort study of PLWH from two large clinical care centers in Munich, Germany.

**Results:**

Of 307 patients with vitamin D deficiency, 124 patients received vitamin D supplementation (weekly supplementation in 84 (67.7%)). 46.4% and 22.5% of patients achieved 25(OH)D levels ≥30 ng/mL after 12 months of weekly and monthly supplementation with cholecalciferol 20,000 IU, respectively (p=0.011). Dosing interval as well as 25(OH)D baseline levels >15 ng/mL were associated with the normalization of 25(OH)D.

**Conclusion:**

A higher rate of 25(OH)D level normalization can be achieved via weekly supplementation. For several PLWH, even a weekly dose of cholecalciferol 20,000 IU might not be adequate to maintain 25(OH)D levels >30 ng/mL without an initial “loading” dose. The response to supplementation is poorly predictable at an individual level.

## 1. Background

Vitamin D deficiency, defined as serum 25-hydroxyvitamin D (25(OH)D) levels <20 ng/mL, is highly prevalent in general and particularly in people living with HIV (PLWH) [1–4]. Although there is a consensus on the possible benefits of vitamin D supplementation in PLWH, a population that is at increased risk of low bone mineral density (BMD) and osteoporosis [5], optimal dosing regimens have not been established. Studies about vitamin D supplementation in HIV-positive populations have been conducted [6–14]. However, indirect comparisons of these studies lead to equivocal conclusions, while direct comparative studies are limited [15, 16]. As HIV infection itself as well as different antiretroviral drugs significantly influence bone, calcium, and vitamin D metabolism [17], it is unclear whether the results of previous studies about the dosing regimens of cholecalciferol supplementation in general populations apply to PLWH.

## 2. Objectives

This study aimed to compare the efficacy of the two different dosing regimens of cholecalciferol supplementation (20,000 IU weekly or monthly) that were used in the clinical routine of two HIV clinical care centers in a retrospective setting in Munich, Germany.

We hypothesized that the use of cholecalciferol 20,000 IU once a week compared to once a month in patients with a baseline 25(OH)D level <20 ng/mL will result in a significantly higher proportion of patients with 25(OH)D levels ≥30 ng/mL after 12 months.

## 3. Methods

The study was performed using a retrospective, longitudinal nested cohort design in two HIV care centers in Munich, Germany. Patients attending the clinics between January 2015 and April 2016 were enrolled in the study if their baseline 25(OH)D was <20 ng/mL and the follow-up data on their 25(OH)D level were available 12 (±2) months after. Patients with elevated serum creatinine levels, indicating impaired renal function, and transgender patients were not included. Data were obtained from the patients' medical records. The primary outcome variable was 25(OH)D after 12 (±2) months of supplementation. The exposure of interest was the dosing strategy of cholecalciferol supplementation. The dosing regimens were cholecalciferol 20,000 IU once a week (center A) and once a month (center B), depending on the different clinical routine standards of the two different centers.

Laboratory parameters were assessed in validated and accredited site-specific local laboratories according to clinical routine standards. The measurement of 25(OH)D level was performed using a chemiluminescence immunoassay (LIAISON, DiaSorin, Italy) in both centers.

Median and interquartile ranges (IQR) or absolute and relative frequencies (%) were used for numeric variables or categorical variables, respectively. The groups were compared using the Mann–Whitney U test for continuous variables and chi-square test for dichotomous variables. To analyze the dichotomous primary endpoint (25(OH)D levels ≥30 ng/mL after 12 months) we aimed at modelling a multivariate regression after a purposeful selection of covariates [18]. Covariates with a p value <0.200 in the univariate regression were considered for multivariate analysis. Dichotomous variables were included without further changes. Continuous variables were categorized into binary variables according to clinically relevant cut-off values (CD4 cell count <350 cells/*μ*L; CD4 cell nadir <200 cells/*μ*L) or after the estimation of the best-discriminating cut-off values after Receiving Operator Characteristics (ROC) analysis (25(OH)D <15 ng/mL at baseline). Results were presented as odds ratio (OR) with 95% confidence interval (95% CI).

The relative risk (RR) and relative risk reduction (1-RR) of the patients after 12 months of vitamin D supplementation were calculated.

A statistical analysis was performed using STATA SE 13.1 software (Stata, College Station, TX, USA). A p value <0.05 was considered statistically significant.

This manuscript was written in accordance with the Strengthening the Reporting of Observational Studies in Epidemiology (STROBE) statement: guidelines for reporting observational studies [19].

## 4. Results

Of the 307 patients with vitamin D deficiency, 252 (82.1%) were men with a median age of 48 (40–54) years. In 124 patients, vitamin D supplementation was initiated after baseline, of which 84 (67.7%) received weekly supplementation. The characteristics of the subgroup are presented in Table 1.

In the site that used weekly supplementation, the percentage of patients that demonstrated 25(OH)D normalization after 12 months was higher among those who received supplementation at 46.4% than those without supplementation at 8.5% (p<0.001). Therefore, the RR of patients with vitamin D deficiency after 12 months of supplementation was 0.18 (0.10–0.32), resulting in a 1-RR of 82% (68%–90%; p<0.001).

In the site that used weekly supplementation, the percentage of patients that demonstrated 25(OH)D normalization after 12 months was higher among those who received supplementation at 22.5% than those without supplementation at 3.3% (p=0.023). The RR of patients with vitamin D deficiency after 12 months of supplementation was 0.15 (0.03–0.78), resulting in a 1-RR of 85% (22%–97%; p=0.023).

No association was observed between suspected interference with vitamin D metabolism and the use of any antiretroviral drugs, such as tenofovir disoproxil fumarate (TDF), efavirenz (EFV), or protease inhibitors (PI) with a pharmacokinetic enhancer, e.g., booster (ritonavir or cobicistat), in our cohort. However, 25(OH)D levels <15 ng/mL (OR: 0.3; 0.1–0.7; p=0.006) as well as a weekly dose of cholecalciferol (OR: 2.7; 1.1–6.4; p=0.029) were significantly associated with the normalization of 25(OH)D levels after 12 months (Table 2). The final overall model (p<0.001) showed a positive predictive value (PPV) and negative predictive value (NPV) of 58.6% and 67.4%, respectively.

In patients with baseline 25(OH)D <15 ng/mL, 25(OH)D was normalized in 10/27 (37.0%) patients with supplementation and in 1/31 (3.2%) patients without supplementation, respectively, in the center that performed weekly supplementation (p=0.001). Meanwhile, 25(OH) was normalized in 4/26 (15.4%) patients in the supplementation group, while it was not normalized in any patient in the group without supplementation in the center that performed monthly dosing (p=0.135).

Stratifying according to baseline levels of 25(OH)D, in patients with severe vitamin D deficiency (< 10 ng/mL) a normalization could be achieved in 10/27 (37.0%) and 4/26 (15.4%) (p=0.074) compared to 29/57 (50.9%) and 5/14 (35.7%) in the group of patients with vitamin D deficiency (p=0.309) following weekly and monthly supplementation, respectively.

## 5. Discussion

The results of previous studies have suggested a U-shaped association between vitamin D concentrations and its effects [20], drawing interest back to dosing regimens of cholecalciferol within the range of recommended daily intake, between 600 IU and 2000 IU [21], with an assumed safe upper limit of 4,0 00 IU [22]. In this analysis, the supplementation strategies represent the lower and upper limit of the recommended daily dose, with about 700 and 2,800 IU, respectively. Thus, we intended to analyze and present real-world data from two cohorts with different supplementation strategies, considering that comparative data on different dosing strategies are limited.

As hypothesized, a weekly dose of cholecalciferol 20,000 IU resulted in a significantly higher proportion of patients who had 25(OH)D levels >30 ng/mL after 12 months: about half of the patients who received weekly supplementation compared to only about a quarter of those who received monthly supplementation achieved 25(OH)D normalization. However, the overall response to vitamin D supplementation seems to be poorly predictable at an individual level, as demonstrated by the poor predictive parameters of our model.

Apart from the dosing regimen, only a low 25(OH)D level at baseline was associated with the normalization of 25(OH)D. In the subgroup of patients with a baseline 25(OH)D level <10 ng/mL, monthly supplementation was not associated with a significantly higher probability of 25(OH)D normalization than without supplementation; weekly supplementation showed a trend towards higher rates of normalization of 25(OH)D levels that failed to achieve statistical significance, most likely due to the low number of patients in this subgroup.

Interestingly, the use of neither efavirenz, nor TDF, nor any boosted PI regimen significantly influenced the response to vitamin D supplementation despite the potential interference with bone and vitamin D metabolism [17, 23–26]. In the absence of HIV-specific factors that seem to influence the response to vitamin D supplementation, as suggested by the results of our data, general recommendations might also apply for PLWH. Therefore, two-step strategies might be more effective: a weekly dose of cholecalciferol 50,000 IU (or 6000 IU daily) for 8 weeks was recommended by the Endocrine Society, followed by a maintenance dose of 1,500–2,000 IU [21]. We cannot, however, state on the role of two-step regimens based on our data, as they were not routinely used in any of the two participating centers. Initial high-dose supplementation followed by low(er) dose maintenance has not been fully implemented in some major guidelines yet. Our data should therefore give rise to questioning single-dose supplementation strategies in PLWH.

However, our study has several limitations, which include the absence of control over adherence to the supplementation. Due to the much higher dose of cholecalciferol in the weekly supplementation group, this group might have been less prone to adherence issues. We do, however, consider these real-life conditions a strength rather than a weakness of our study, as adherence issues are a problem that has to be considered in patients outside of clinical studies. Our data therefore indicate that a higher-dosing of cholecalciferol might possibly account for potential adherence problem, while at the same time we did not observe toxic concentrations. Also, we cannot provide data on nutritional intake of vitamin D and calcium available from clinical routine. In addition, the use of the nested retrospective design resulted in considerable imbalances in the group characteristics that were adjusted at an analytic level.

Furthermore, we cannot provide further (surrogate) markers of interest such as parathyroid hormone or bone turnover markers or data on the endpoints, such as changes in BMD, fractures, or nonskeletal health effects, which are more relevant than the 25(OH)D levels alone. Because vitamin D supplementation is often guided by 25(OH)D levels in routine patient care, we still believe that the results of our study are important in clinical practice. Lastly, although the sample size of the present study is reasonable, particularly when compared to that of other studies in PLWH, it is still relatively small, which is indicated by wide confidence intervals and resulted in point estimates that were not highly robust. The smaller sample size of the monthly supplementation group therefore has to be kept in mind and particularly for the analysis of an ethnic influence of the success of supplementation as well as the subgroup analysis of baseline deficiency and severe deficiency; the low number of patients in the respective groups has resulted in a power too low to detect a “real” association. As 25(OH)D levels are known to be highly seasonably, which is also applicable to PLWH as demonstrated in our own cohort [27], one might argue that we did not adjust for seasonal differences in both groups adequately. This is particularly true for the absolute values of 25(OH). However, for our follow-up we chose a 12-month interval to guarantee same season at baseline and the end of the follow-up, which should exclude relevant confounding by season for our most important endpoints that were related to the changes of 25(OH)D levels after initiation of supplementation rather than absolute values.

## 6. Conclusions

A monthly dose of cholecalciferol 20,000 IU (corresponding to approximately 700 IU per day) might not be adequate to normalize serum 25(OH)D levels in most PLWH with vitamin D insufficiency, particularly when the baseline levels are <15 ng/mL. Because a cumulative dose of more than 80,000 IU per month (approximately 2,900 IU per day) led to the normalization of 25(OH)D level in only about 50% of patients, we encourage evaluating other strategies in PLWH, particularly all two-step supplementation regimens with a high-dose “loading,” followed by a lower maintenance dose, as recommended for the general population [21]. HIV-specific factors do not seem to affect the response to cholecalciferol supplementation relevantly. Therefore, supplementation strategies as developed from data of non-PWLH-populations also seem to be applicable for PLWH.

## Figures and Tables

**Table 1:** Characteristics of the subgroup per supplementation regimen at baseline and after 12 months of supplementation ([S] = serum concentration).

		Unit	Weekly	Monthly	P value
			(N=84)	(N=40)	
	***Age***	*years (IQR)*	48 (40–57)	46 (39–55)	0.234
	***Gender (male)***	*N (%)*	67 (79.8)	31 (77.5)	0.772
	***Ethnicity (African)***	*N (%)*	9 (10.7)	8 (20.0)	0.160
	***Creatinine [S]***	*mg/dL (IQR)*	0.9 (0.8–1.1)	0.9 (0.7–1.1)	0.212
	***Viral load***	*copies/mL*	19 (19–19)	283 (19–8,793)	**<0.001**
	***Viral load <200/mL***	*N (%)*	81 (96.4)	20 (50.0)	**<0.001**
	***CD4 cell count (absolute)***	*cells/μL (IQR)*	632 (496–861)	395 (183–610)	**<0.001**
	***CD4 cell count (relative)***	*% (IQR)*	31.5 (24.0–37.0)	21.5 (11.3–32.0)	**<0.001**
	*Missing data *	*N (%)*	-	-	
	***HIV risk group: MSM***	*N (%)*	49 (48.3)	11 (27.5)	**0.001**

*ART AT BASELINE*	***Protease Inhibitor-based ART Regimen***	*N (%)*	33 (39.3)	13 (32.5)	0.465
	***Efavirenz-based ART***	N (%)	15 (17.9)	9 (22.5)	0.541
	***Tenofovir disoproxil fumarate-based ART***	N (%)	52 (61.9)	27 (67.5)	0.545

*BASELINE*	***25(OH)D [S]***	*ng/mL (IQR)*	12 (9–16)	8 (5–13)	**<0.001**
	***25(OH)D <10 ng/dL***	*N (%)*	27 (32.1)	26 (65.0)	**0.001**
	*Missing data *	*N (%)*	-	-	
	***Calcium [S]***	*mg/dL (IQR)*	2.26 (2.18–2.32)	2.31 (2.26–2.39)	**0.020**
	*Missing data *	*N (%)*	-	17 (42.5%)	
	***Phosphate [S]***	*mg/dL (IQR)*	3.0 (2.7–3.4)	3.3 (3.0–3.8)	**0.019**
	*Missing data *	*N (%)*	-	5 (12.5%)	
	***AP [S]***	*IU/mL (IQR)*	88 (72–108)	84 (60–103)	0.205
	*Missing data *	*N (%)*	-	-	

*AFTER 12 MONTHS*	***25(OH)D [S]***	*ng/mL (IQR)*	28 (21–36)	24 (18–30)	**0.004**
	***25(OH)D ≥30 ng/mL***	*N (%)*	39 (46.4)	9 (22.5)	**0.011**
	***Calcium [S]***	*mg/dL (IQR)*	2.28 (2.23–2.35)	2.39 (2.35–2.48)	**<0.001**
	*Missing data *	*N (%)*	-	5 (12.5%)	
	***Phosphate [S]***	*mg/dL (IQR)*	3.2 (2.8–3.6)	3.3 (3.0–3.7)	0.122
	*Missing data *	*N (%)*	-	2 (5.0%)	
	***AP [S]***	*IU/mL (IQR)*	85 (70–104)	78 (67–100)	0.211
	*Missing data *	*N (%)*	-	-	

**Table 2:** Results of the univariate and multivariate regression analysis on the association between a 25(OH)D serum level of ≥30 ng/mL and supplementation after 12 months.

	Univariate		Multivariate	
	OR (95% CI)	p value	OR (95% CI)	p value
Weekly dose of supplementation	3.0 (1.3–7.0)	**0.012**	2.7 (1.1–6.4)	**0.029**
Gender (male)	1.2 (0.5–2.9)	0.672		
Age	1.0 (0.9–1.1)	0.284		
Ethnicity (African)	0.6 (0.2–1.9)	0.400		
Baseline 25(OH)D (<15 ng/mL)	0.3 (0.1–0.6)	**0.002**	0.3 (0.1–0.7)	**0.006**
Baseline Viral load (<200/mL)	2.0 (0.7–5.5)	0.174		
Baseline CD4 cell count (<350/*μ*L)	0.5 (0.2–1.3)	0.131		
CD4 nadir < 200 cells/*μ*L	0.7 (0.3-1.5)	0.390		
HIV risk group: MSM	1.0 (0.5-2.0)	0.934		
Time since diagnosis (years)	1.0 (0.9-1.1)	0.317		
Use of tenofovir disoproxil fumarate (TDF)	0.6 (0.3–1.3)	0.171		
Use of efavirenz (EFV)	0.5 (0.2–1.3)	0.131		
Use of boosted protease inhibitor (PI)	1.6 (0.8–3.3)	0.224		
cART including at least two of the following: TDF, EFV, boosted PI	0.9 (0.4-1.8)	0.715		

## Data Availability

The data used to support the findings of this study are available from the corresponding author upon request.

## Abbreviations

25(OH)D: 25-Hydroxyvitamin D
AP: Alkaline phosphatase
ART: Antiretroviral therapy
EFV: Efavirenz
IQR: Interquartile range
NPV: Negative predictive value
OR: Odds ratio
PI: Protease inhibitor
PLWH: People living with HIV
PPV: Positive predictive value
RR: Relative risk
TDF: Tenofovir disoproxil fumarate.
